# Supplementary material for: Formal recognition and classification of gene transfer agents as viriforms
Source: Virus Evol. 2022 Oct 15;8(2):veac100. doi: 10.1093/ve/veac100 (PMC9662315; doi:10.1093/ve/veac100)
Supplement: veac100_Supp [file veac100_supp.zip › Supplementary_Tables_S1_S6.pdf]

Supplementary Tables S1–S6

for

Formal Recognition and Classification of Gene Transfer Agents as Viriforms

Roman Kogay[1], Sonja Koppenhöfer[2], J. Thomas Beatty[3], Jens H. Kuhn[4], Andrew S. Lang[2], and Olga Zhaxybayeva[1,#]

[1]Department of Biological Sciences, Dartmouth College, Hanover, NH, USA; [2]Department of Biology, Memorial University of Newfoundland, St. John's, NL, Canada; [3]Department of Microbiology & Immunology, University of British Columbia, Vancouver, BC, Canada; [4]Integrated Research Facility at Fort Detrick, Division of Clinical Research, National Institute of Allergy and Infectious Diseases, National Institutes of Health, Frederick, MD, USA

[#]Correspondence to Olga Zhaxybayeva, olga.zhaxybayeva@dartmouth.edu

Supplementary Table S1. Rhodobacter capsulatus gene transfer agent (RcGTA) genes, their locus tags, and their functional annotations in the host (*Rhodobacter capsulatus* SB1003) genome (GenBank accession CP001312.1).

Locus tag	Start	End	Strand	GenBank annotation*
RCAP_rcc00171	203033	204148	+	tail fiber protein [1]
RCAP_rcc00555	593866	594477	+	holin-associated N-acetylmuramidase
RCAP_rcc00556	594468	594962	+	holin family protein
RCAP_rcc01079	1149611	1149865	+	*ghsA* head spike protein [2]
RCAP_rcc01080	1149862	1150839	+	*ghsB* head spike protein [2]
RCAP_rcc01682	1818689	1819012	+	small terminase [3]
RCAP_rcc01683	1818963	1820309	+	terminase family protein
RCAP_rcc01684	1820495	1821685	+	phage portal protein
RCAP_rcc01685	1821695	1821922	+	hypothetical protein
RCAP_rcc01686	1821933	1822487	+	HK97 family phage prohead protease
RCAP_rcc01687	1822519	1823715	+	phage major capsid protein
RCAP_rcc01688	1823888	1824481	+	adaptor protein [4]
RCAP_rcc01689	1824478	1824816	+	head-tail adaptor protein
RCAP_rcc01690	1824813	1825220	+	tail terminator protein [4]
RCAP_rcc01691	1825261	1825674	+	phage major tail protein, TP901-1 family
RCAP_rcc01692	1825682	1826008	+	gene transfer agent family protein
RCAP_rcc01693	1826005	1826232	+	phage tail assembly chaperone
RCAP_rcc01694	1826219	1826878	+	phage tail tape measure protein
RCAP_rcc01695	1826889	1827521	+	distal tail protein [4]
RCAP_rcc01696	1827521	1828411	+	baseplate hub protein [4]
RCAP_rcc01697	1828408	1828860	+	peptidase
RCAP_rcc01698	1828861	1832775	+	glycoside hydrolase/phage tail family protein
RCAP_rcc01865	2018987	2020135	-	*gafA* transcriptional regulator [5]
RCAP_rcc01866	2020352	2020660	+	Possibly, maturation/assembly protein [1]

* For some genes, the GenBank annotations in the host genome record were replaced with more informative functional annotations from experimental work, with relevant citations provided.

1. Hynes, A.P., Shakya, M., Mercer, R.G., Grull, M.P., Bown, L., Davidson, F., Steffen, E., Matchem, H., Peach, M.E., Berger, T., Grebe, K., Zhaxybayeva, O., and Lang, A.S. Functional and evolutionary characterization of a gene transfer agent's multilocus "genome". *Mol Biol Evol*, 2016. **33**(10): 2530-43.
2. Westbye, A.B., Kuchinski, K., Yip, C.K., and Beatty, J.T. The Gene Transfer Agent RcGTA contains head spikes needed for binding to the *Rhodobacter capsulatus* polysaccharide cell capsule. *J Mol Biol*, 2016. **428**(2 Pt B): 477-91.
3. Sherlock, D., Leong, J.X., and Fogg, P.C.M. Identification of the first gene transfer agent (GTA) small terminase in *Rhodobacter capsulatus* and its role in GTA production and packaging of DNA. *J Virol*, 2019. **93**(23): e01328-19.
4. Bardy, P., Fuzik, T., Hrebik, D., Pantucek, R., Thomas Beatty, J., and Plevka, P. Structure and mechanism of DNA delivery of a gene transfer agent. *Nat Commun*, 2020. **11**(1): 3034.
5. Fogg, P.C.M. Identification and characterization of a direct activator of a gene transfer agent. *Nat Commun*, 2019. **10**(1): 595.

Supplementary Table S2. Dinoroseobacter shibae gene transfer agent (DsGTA) genes, their locus tags, and their functional annotations in the host (*Dinoroseobacter shibae* DFL12) genome (GenBank accession CP000830.1).

Locus tag	Start	End	Strand	GenBank annotation
Dshi_1584	1649803	1650927	-	DUF6456 domain-containing protein
Dshi_1585	1651121	1651423	+	DUF6477 family protein
Dshi_1756	1820645	1821166	-	holin family protein
Dshi_1757	1821157	1821768	-	holin-associated N-acetylmuramidase
Dshi_2162	2291686	2292393	-	DUF2793 domain-containing protein
Dshi_2163	2292386	2296303	-	glycoside hydrolase/phage tail family protein
Dshi_2164	2296307	2296747	-	peptidase
Dshi_2165	2296744	2297634	-	DUF2163 domain-containing protein
Dshi_2166	2297631	2298263	-	DUF2460 domain-containing protein
Dshi_2167	2298277	2298945	-	phage tail tape measure protein
Dshi_2168	2298929	2299144	-	phage tail assembly chaperone
Dshi_2169	2299141	2299470	-	gene transfer agent family protein
Dshi_2170	2299476	2299889	-	phage major tail protein, TP901-1 family
Dshi_2171	2299911	2300321	-	DUF3168 domain-containing protein
Dshi_2172	2300318	2300656	-	head-tail adaptor protein
Dshi_2173	2300653	2301243	-	hypothetical protein
Dshi_2174	2301398	2302615	-	phage major capsid protein
Dshi_2175	2302612	2303229	-	HK97 family phage prohead protease
Dshi_2176	2303242	2303502	-	hypothetical protein
Dshi_2177	2303499	2304689	-	phage portal protein
Dshi_2178	2304797	2306161	-	terminase family protein

Supplementary Table S3. Rhodovulum sulfidophilum gene transfer agent (RsGTA) genes, their locus tags, and their functional annotations in the host (*Rhodovulum sulfidophilum* DSM 1374) genome (GenBank accession CP015418.1).

Locus tag	Start	End	Strand	GenBank annotation
A6W98_RS21760	181175	182050	+	DUF2793 domain-containing protein
A6W98_09125	1925273	1925629	+	hypothetical protein
A6W98_09130	1925622	1926899	+	terminase family protein
A6W98_09135	1927031	1928206	+	phage portal protein
A6W98_09140	1928209	1928451	+	hypothetical protein
A6W98_09145	1928472	1929038	+	HK97 family phage prohead protease
A6W98_09150	1929082	1930269	+	phage major capsid protein
A6W98_09155	1930432	1931031	+	hypothetical protein
A6W98_09160	1931028	1931369	+	head-tail adaptor protein
A6W98_09165	1931366	1931776	+	DUF3168 domain-containing protein
A6W98_09170	1931800	1932213	+	phage major tail protein, TP901-1 family
A6W98_09175	1932216	1932548	+	gene transfer agent family protein
A6W98_09180	1932538	1932732	+	phage tail assembly chaperone
A6W98_09185	1932736	1933404	+	phage tail tape measure protein
A6W98_09190	1933420	1934052	+	DUF2460 domain-containing protein
A6W98_09195	1934052	1934939	+	DUF2163 domain-containing protein
A6W98_09200	1934936	1935379	+	NlpC/P60 family protein
A6W98_09205	1935383	1939282	+	glycoside hydrolase/phage tail family protein
A6W98_10845	2293061	2293672	+	holin-associated N-acetylmuramidase
A6W98_10850	2293663	2294229	+	holin family protein
A6W98_12585	2663257	2663559	-	DUF6477 family protein
A6W98_12590	2663793	2664887	+	DUF6456 domain-containing protein

Supplementary Table S4. Ruegeria pomeroyi gene transfer agent (RpGTA) genes, their locus tags, and their functional annotations in the host (*Ruegeria pomeroyi* DSS-3) genome (GenBank accession CP000031.2).

Locus tag	Start	End	Strand	GenBank annotation
SPO1878	2001541	2002152	+	holin-associated N-acetylmuramidase
SPO_RS09550	2002143	2002721	+	holin family protein
SPO2106	2242168	2243322	-	DUF6456 domain-containing protein
SPO2107	2243566	2243874	+	DUF6477 family protein
SPO2250	2393488	2397414	-	glycoside hydrolase/phage tail family protein
SPO2251	2397414	2397860	-	peptidase
SPO2252	2397857	2398744	-	DUF2163 domain-containing protein
SPO2253	2398744	2399376	-	DUF2460 domain-containing protein
SPO2254	2399392	2400054	-	phage tail tape measure protein
SPO2255	2400041	2400250	-	phage tail assembly chaperone
SPO2256	2400247	2400567	-	gene transfer agent family protein
SPO2257	2400570	2400983	-	phage major tail protein, TP901-1 family
SPO2258	2401008	2401421	-	DUF3168 domain-containing protein
SPO2259	2401418	2401756	-	phage head closure protein
SPO2260	2401753	2402352	-	head-tail connector protein
SPO2261	2402517	2403695	-	phage major capsid protein
SPO2262	2403729	2404286	-	HK97 family phage prohead protease
SPO2263	2404315	2404539	-	hypothetical protein
SPO2264	2404532	2405704	-	phage portal protein
SPO2266	2405830	2407056	-	terminase family protein
SPO_RS11495	2407109	2407453	-	hypothetical protein
SPO2726	2911999	2913924	-	DUF2793 domain-containing protein

Supplementary Table S5. Bartonella gene transfer agent (BaGTA) genes, their locus tags, and their functional annotations in the host (*Bartonella henselae* str. Houston-1) genome (GenBank accession BX897699.1).

Locus tag	Start	End	Strand	GenBank annotation*
BH13940	1602076	1602738	-	phage related lysozyme
BH13950	1603017	1603283	-	hypothetical protein
BH13960	1604763	1605770	-	phage tail collar protein [1]
BH13970	1605767	1606891	-	peptidase, chaperon [1]
BH13990	1607638	1609101	-	hypothetical protein
BH14000	1610017	1610712	-	hypothetical protein
BH14010	1611092	1612210	-	major capsid protein [1]
BH14040	1613196	1613993	-	hypothetical protein
BH14060	1614966	1616819	-	hypothetical protein
BH14070	1616804	1618012	-	phage terminase [1]
BH14090	1618545	1618874	-	hypothetical protein
BH14150	1625365	1626306	+	hypothetical protein
BH14200	1630937	1631227	+	hypothetical protein
BH14220	1631403	1632176	+	hypothetical protein
BH14240	1632865	1633209	-	hypothetical protein
BH14310	1638603	1638968	+	hypothetical protein
BH14320	1639193	1639567	+	hypothetical protein
BH14330	1639687	1639965	+	hypothetical protein
BH14470	1655075	1655464	-	hypothetical protein
BH14480	1655778	1656095	-	hypothetical protein
BH14490	1656650	1657579	+	hypothetical protein
BH14500	1657576	1657869	+	hypothetical protein
BH14510	1657853	1658998	+	hypothetical protein
BH14520	1658982	1661741	+	phage related protein
BH14530	1663283	1663849	+	hypothetical protein
BH14580	1676092	1676691	+	hypothetical protein
BH14600	1679632	1680294	+	phage related lysozyme
BH14610	1680291	1680518	+	hypothetical protein
BH14620	1680647	1680868	+	hypothetical protein

* For some genes, the GenBank annotations in the host genome record were replaced with more informative functional annotations from experimental work, with relevant citations provided.

1. Quebatte, M., Christen, M., Harms, A., Korner, J., Christen, B., and Dehio, C. Gene transfer agent promotes evolvability within the fittest subpopulation of a bacterial pathogen. *Cell Syst*, 2017. 4(6): 611-621.e6.

Supplementary Table S6. Brachyspira hyodysenteriae gene transfer agent (BhGTA) genes, their locus tags, and their functional annotations in the host (*Brachyspira hyodysenteriae* WA1) genome (GenBank accession CP001357.1).

Locus tag	Start	End	Strand	GenBank annotation*
BHWA1_01838	2121760	2123001	+	hypothetical protein
BHWA1_01839	2123304	2124257	+	hypothetical protein
BHWA1_01840	2124448	2124816	+	hypothetical protein
BHWA1_01841	2125122	2126165	+	major capsid protein [1]
BHWA1_01844	2127442	2127999	+	hypothetical protein
BHWA1_01846	2128455	2129852	+	hypothetical protein
BHWA1_01850	2131660	2132535	+	hypothetical protein
BHWA1_01851	2132609	2135149	+	hypothetical protein
BHWA1_01852	2135161	2135709	+	hypothetical protein
BHWA1_01853	2135714	2136307	+	endolysin [1]
BHWA1_01855	2137181	2137438	+	holin [1]

* For some genes, the GenBank annotations in the host genome record were replaced with more informative functional annotations from experimental work, with relevant citations provided.

1. Matson, E.G., Thompson, M.G., Humphrey, S.B., Zuerner, R.L., and Stanton, T.B. Identification of genes of VSH-1, a prophage-like gene transfer agent of *Brachyspira hyodysenteriae. J Bacteriol*, 2005. **187**(17): 5885-92.
